# GMIEC: a shiny application for the identification of gene-targeted drugs for precision medicine

**DOI:** 10.1186/s12864-020-06996-y

**Published:** 2020-09-10

**Authors:** Guidantonio Malagoli Tagliazucchi, Cristian Taccioli

**Affiliations:** 1 Division of Cardiology, Azienda Ospedaliero-Universitaria di Parma, 43126 Parma, Italy; 2 Present address: Department of Genetics, Evolution and Environment, Darwin Building, Gower Street WC1E 6BT, London, UK; 3 Department of Animal Medicine, Production and Health, University of Padova, 35020 Legnaro, PD Italy

**Keywords:** Medical genomics, Precision medicine, Genomics, R, Shiny

## Abstract

**Abstract:**

**Background:**

Precision medicine is a medical approach that takes into account individual genetic variability and often requires Next Generation Sequencing data in order to predict new treatments. Here we present GMIEC, *Genomic Modules Identification et Characterization for genomics medicine,* an application that is able to identify specific drugs at the level of single patient integrating multi-omics data such as RNA-sequencing, copy-number variation, methylation, Chromatin Immuno-Precipitation and Exome/Whole Genome sequencing. It is also possible to include clinical data related to each patient. GMIEC has been developed as a web-based R-Shiny platform and gives as output a table easy to use and explore.

**Results:**

We present GMIEC, a Shiny application for genomics medicine. The tool allows the users the integration of two or more multiple omics datasets (e.g. gene-expression, copy-number), at sample level, to identify groups of genes that share common genomic and corresponding drugs. We demonstrate the characteristics of our application by using it to analyze a prostate cancer data set.

**Conclusions:**

GMIEC provides a simple interface for genomics medicine. GMIEC was develop with Shiny to provide an application that does not require advanced programming skills. GMIEC consists of three sub-application for the analysis (GMIEC-AN), the visualization (GMIEC-VIS) and the exploration of results (GMIEC-RES). GMIEC is an open source software and is available at https://github.com/guidmt/GMIEC-shiny

## Background

More efficient drug therapies and better treatment are the promises of personalized medicine. In fact, precision medicine has created innovative opportunities to identify new drugs and therapeutic strategies. The integration of Next Generation Sequencing (NGS) data can be an important support to further tailor health care for each patient. Several methods of data fusion exist [1] and many tools were developed to integrate multiple genomic information [2–8]. However, these methods are not always straightforward because do not provide graphical interfaces and the possibility to upload custom datasets. Moreover, it is not possible to search for group of genes (modules) with common molecular profiles as in GMIEC (*Genomic Modules Identification et Characterization for genomics medicine)*. GMIEC is a flexible application that allows to integrate gene-expression, copy-number variation, methylation, Chromatin Immuno-Precipitation sequencing, mutations, and clinical data at the level of single patient. It analyses and, successively, links each module with the corresponding drugs, a feature able to identify new therapeutic targets. Furthermore, GMIEC was developed not only for cancer research but can also be used for other diseases (e.g. diabetes and genetic disorders). GMIEC was developed as an application that does not require advanced programming skills (see section “Installation and Usage” and “Prerequisite” Additional file 1 for details about the installation). In fact, it is a web-based R-Shiny tool (www.rstudio.com/shiny) that helps the creation of interactive web pages that can be queried through the use of buttons.

## Implementation

### GMIEC framework

**Fig. 1:**
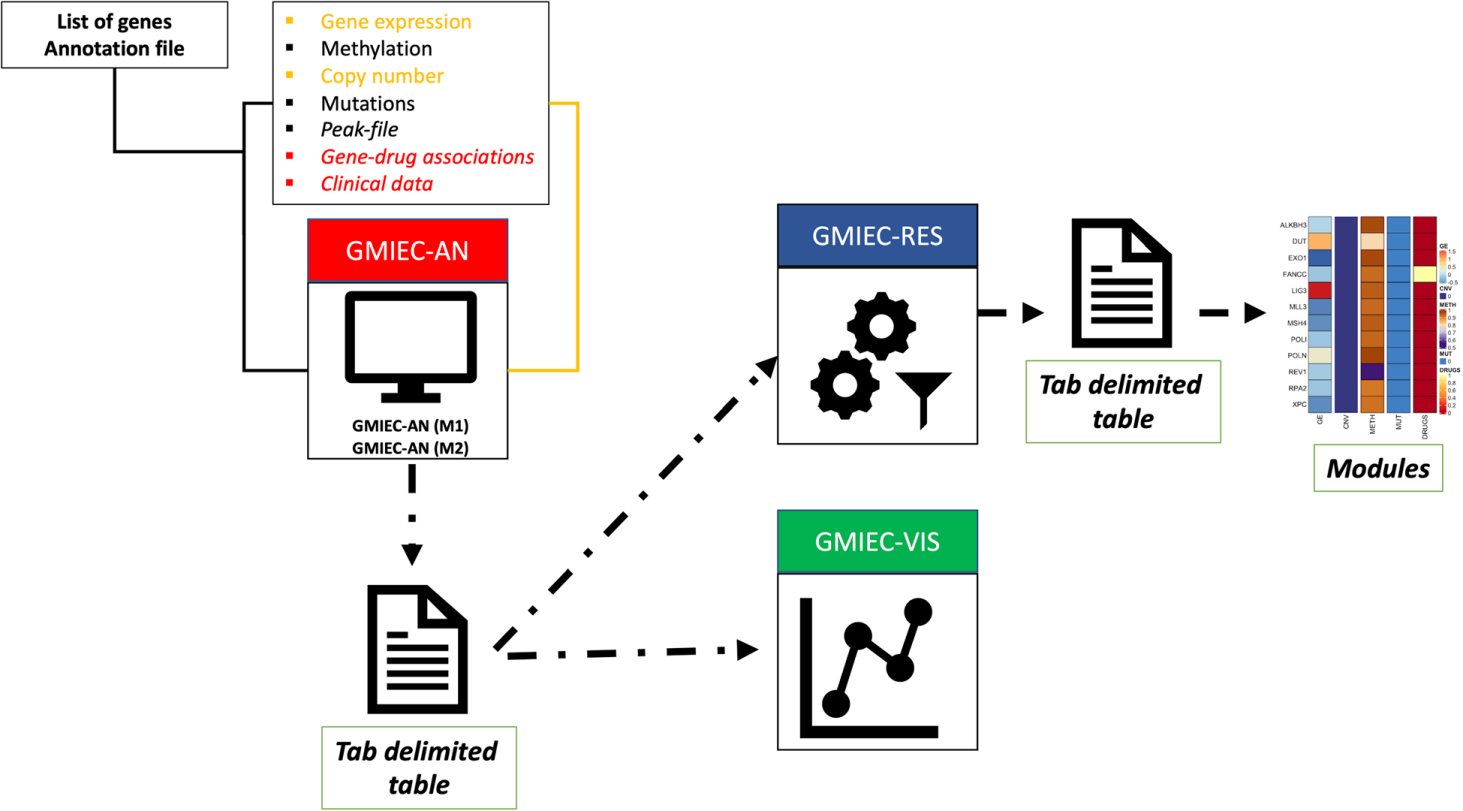
GMIEC framework of analysis. In the first step, the user uploads the omics datasets using a gui-interface. The main application of GMIEC, GMIEC-AN implements two methods of analysis (M1, M2). GMIEC-AN allows to analyze two (orange) or more datasets. When the user provides a file with the genomic coordinates (e.g. bed file), an annotation file (e.g. RefSeq) is also required. The only mandatory file for GMIEC is a file with the association genes – drugs (red text); users can personalize it. The output of GMIEC-AN is a tab delimited file containing the genes and drugs modules identified during the analysis. This output can be upload into GMIE-VIS to explore the results through dynamic tables. Otherwise, the user can upload the output of GMIEC-AN on GMIEC-results

GMIEC consists of three sub-applications (Fig. 1): i) *GMIEC-AN* is used for the analysis of a minimum of two datasets; ii) *GMIEC-results* parses the output of *GMIEC-AN*, performs the automatic selection of the modules and outputs the results; iii) *GMIEC-VIS*, is a tool that helps to explore the results of *GMIEC-AN*. GMIEC was developed to analyze datasets with different sizes. In fact, the user can perform one analysis considering gene-expression, copy-number variation, methylation and mutation data and using only two or more of this kind of data. The only mandatory file that is required to run one analysis is a table with the associations between the genes and drugs (highlighted in red). The main output of GMIEC is a tab-delimitated file (green box), in which the rows are the samples and columns the modules. This file can be used for downstream analysis or uploaded on GMIEC-results or GMIEC-vis. GMIEC-results is a sub-application that performs the automatic selection of the modules in each patient using the output from the analysis with GMIEC-AN. This simplified version of the output file can be also uploaded on GMIEC-results (blue arrow) to visualize the levels of the gene-expression, methylation and copy number variation, mutations (or the omics data provided) of the genes inside one module of a specific patient. GMIEC-VIS provides functionalities to explore the results of GMIEC-AN using a dynamic interface with heatmaps, charts and tables.

GMIEC is an open source software and is available at https://github.com/guidmt/GMIEC-shiny, its documentation is available at https://github.com/guidmt/GMIEC-shiny/wiki.

### Methodology

GMIEC-AN implements two strategies of analysis (called M1 and M2), (see section “Computational Methods” Additional file 1 and Supplementary Table S1, for a complete description of all methods). The analytical procedure implemented in M1 in the first step, selects only the profiles of the genomics data of a given set of genes G (see section “GMIEC framework of analysis” Additional File 1 and Fig. S1). This gene-set can be provided by the user. For example, the user can upload a list of genes of interest. Then, a vector I = {i_1_, i_2_, i_3_, i_i_}, that consists of *i* individuals in all genomic data, will be created. GMIEC-AN, iterates on each *i*-th subject in I and selects the genomic profiles here defined as *m* (e.g. gene-expression, copy-number) for the current *i*. The result of this step is a matrix M_*i*_ = (G x m). For each patient, the number of rows and columns of this matrix correspond respectively to the number of genes in G and the number of datasets uploaded by the user. Therefore, each column of M contains the values of each genomic dataset (e.g. gene-expression, copy-number alteration). Then, each M_*i*_, is used to train an unsupervised random forest algorithm (randomForest R-package) to compute the proximity matrix. The proximity matrix is a symmetric matrix in which each value is the proportion of times that each pair of genes resides on the same terminal node of the tree. Then, the matrix is used as input for a k-means method to identify clusters of genes (step 5, see section “GMIEC framework of analysis” Additional file 1, Fig. S1) with a pre-defined number of clusters chosen by the user. GMIEC-AN can also determine the optimal number of clusters using silhouette analysis. Each cluster will contain genes that share common genomic features that we define as gene modules (GMs). Therefore, each GM is merged with a file containing the association between the genes and the drugs (step 6, see section “GMIEC framework of analysis” Additional file 1, Fig. S1). The user can create a custom file containing the associations between specific genes and drugs, or download gene/drugs datasets available online [9]. In the final step, the scores are computed (step 7, see section “GMIEC framework of analysis” Additional file 1, Fig. S1) for the genomic alterations and the fraction of drugs identified in a module. The results of this analysis are, then, merged with the clinical data provided. The M2 strategy (logic approach and k-modes) implement a different analytical procedure. For each patient, we applied logic rules to map if the genes of a patient share or not some genomic features (e.g. copy-number variation). To this aim, we used a similar approach based on fuzzy logic used [10]. In our context we define different criteria. For all genes (*g*) of a patient (*i)* a matrix M_i_ = (g x r), in which n are the genes and r the rules, is generated. If a gene respect a given rule, this is marked as “1”, otherwise is marked as “0” (see section “Logic approach and k-modes” Additional file 1). The logic rules implemented are the same if the user upload more or two datasets. However, in the last case, the rules that will be applied, depends on the types of dataset provided by the users (e.g. gene-expression and copy-number variation), only the rules useful for a specific dataset will be applied. After that the analysis for one patient is completed, the resulting matrix (M_i_) is used as input for k-modes [11] to group the genes with common properties (copy number alterations, over-expression etc). The number of clusters to use in k-modes is defined by the user.

## Results

### Comparison with other resources available

Several tools and databases have been developed during the last years with the aim to facilitate the integration and analysis of multi-omics data in the field of the personalized medicine. Here we report a description of these resources and the comparisons with GMIEC (see Table 1). DriverDBv3 [12] is a database that integrates somatic mutations, RNA expression, miRNA expression, methylation, copy number variation and clinical data to identify driver genes in cancers. Users can query the database providing the name of a gene and the type of a cancer. The database implements an interface to compute the survival probabilities of the patients between cancers. PanDrugs [8], is a database with drugs-targets associations. The input files for this database are a vcf table and a ranked gene list respectively. The user can query the database using the name of a gene or drug. DriverDBv3 and PanDrugs allows the user to use the databases with multiple options. However, their flexibility to receive as input multiple data-sets is limited. Other two databases PreMedKB [6] and OncoKB [13] does not allow the user to upload their datasets. Notably, Cancer Variant Explorer [19] is a shiny and R-package that perform the prioritization of the variants to identify mechanisms that drives the resistance and the druggability of a treatment. Users can provide as input genetic variants annotated with Oncotar [14]. IndividPath [15] is a R-package that analyze gene-expression data to identify de-regulated pathways in individual disease samples. Differently from GMIEC, these tools do not give the opportunity to analyze multiple kinds of data or lack of a GUI interface. iPAS [16] is a R-package that identify de-regulated pathways in patient-specific manner using groups of patients (e.g. disease and healthy). This tool does not offer the opportunity to analyze multi-omics data and it is not available a GUI interface. Prodigy [18] is an algorithm for patient-specific ranking of genes. This tool requires as input expression and variants data, protein-protein interactions data. The tool was developed as an R-package and a GUI interface is not available, moreover is not possible provide as input other kinds of data (e.g. copy number alterations). Finally, PharmacoGx [17] is a tool that allows to download and interrogate large pharmacogenomic datasets [20, 21]. GMIEC, therefore, presents some features that are not presents in the existing tools. GMIEC not only provides a GUI interface based on Shiny, but can analyze multi-omics datasets in flexible way.

**Table 1:** Comparison between GMIEC and others bioinformatic tools available in precision medicine

Name resource	Type application	Uploading custom data	Input file	GUI interface	Reference
**GMIEC**	Web-based/ shiny application	yes	Gene-expression, copy number, methylation, table with genes mutated, drugs-genes file, clinical data, gene-list, bed file.	yes	**–**
**DriverDBv3**	Web-based	no	The web interface incorporates somatic mutations, RNA-seq, miRNA-expression, methylation, copy number, clinical data.	yes	[12]
**OncoKB**	Web-based	no	Users can query the database gene, alteration, drug	yes	[13]
**PanDrugs**	Web-based	yes	VCF, RNK, gene-lists, drug query	yes	[8]
**PreMedKB**	Web-based	no	Users can query the resource for variant, disease, drug, gene or combinations of these category.	yes	[6]
**Cancer Variant Explorer**	R-package	yes	Clinical, mutation	no	[14]
**IndividPath**	R-package	yes	Gene-expression	no	[15]
**iPAS**	R-package	yes	Gene-expression	no	[16]
**PharmacoGx**	R-package	no	Pharmacogenomic datasets	no	[17]
**Prodigy**	R-package	yes	Protein-protein interaction network, gene-expression, mutations	no	[18]

Table 1. Comparison the bioinformatics tools and databases that can be used in the context of precision medicine. The column “name resource” contains the name of the software/database. The second column (“Type application”) described if a tool is a R-package or a web-based application. For each tool, the input file required for the analysis are reported in column (“Input file”). The column “GUI interface” highlight if a given tool/database was implemented with a GUI interface or not.

### Input and output

The input files required by GMIEC are:

I. **omics data**: gene-expression, copy-number, epigenetics and mutations data in the form of tab-delimited files. In the case the genetic variants data, the user might create a file with only the mutations of interest (e.g. with deleterious variants). GMIEC receive as input matrices with the genomic data obtained from standard pipeline of analysis.
II. **drugs-genes table**: a tab delimited file with the association between genes and drugs. The user can create a custom file selecting only the drugs and genes that are important for the disease of interest or download them from our repository.
III. **clinical data**: an optional tab delimited file with clinical variables to include in the analysis.
IV. **list of genes**: a list of genes of interest (e.g. genes of a pathway). The list of genes can be directly uploaded by the user. Otherwise if the user provides a file with genomic coordinates, the list of genes is obtained through an annotation step.

The principal output of GMIEC are table delimited files that can be easily opened with Microsoft Excel or Libre/Open Office. This output contains the gene modules (gene groups or GMs) and drug modules (drug groups or DMs) identified during the analysis. The output reports also columns containing the scores (see section “Computation of the scores” Additional file 1) that are a quantification of the level of genomic alterations of the genes in GM, and the fraction of drugs linked to specific GMs. GMIEC allows also to generate a dynamic report (see Fig. 1) that can be interrogated directly through a GUI interface, GMIEC-VIS. GMIEC-VIS allows to visualize, for each patient, tables with statistics related to the gene modules, and the lists of drugs and genes identified. GMIEC-results is another sub-application that allows to parse the output of GMIEC and automatically select a module for patient. GMIEC-results can be also used to visualize the levels the omics data for the genes inside each module.

### GMIEC interface

**Fig. 2:**
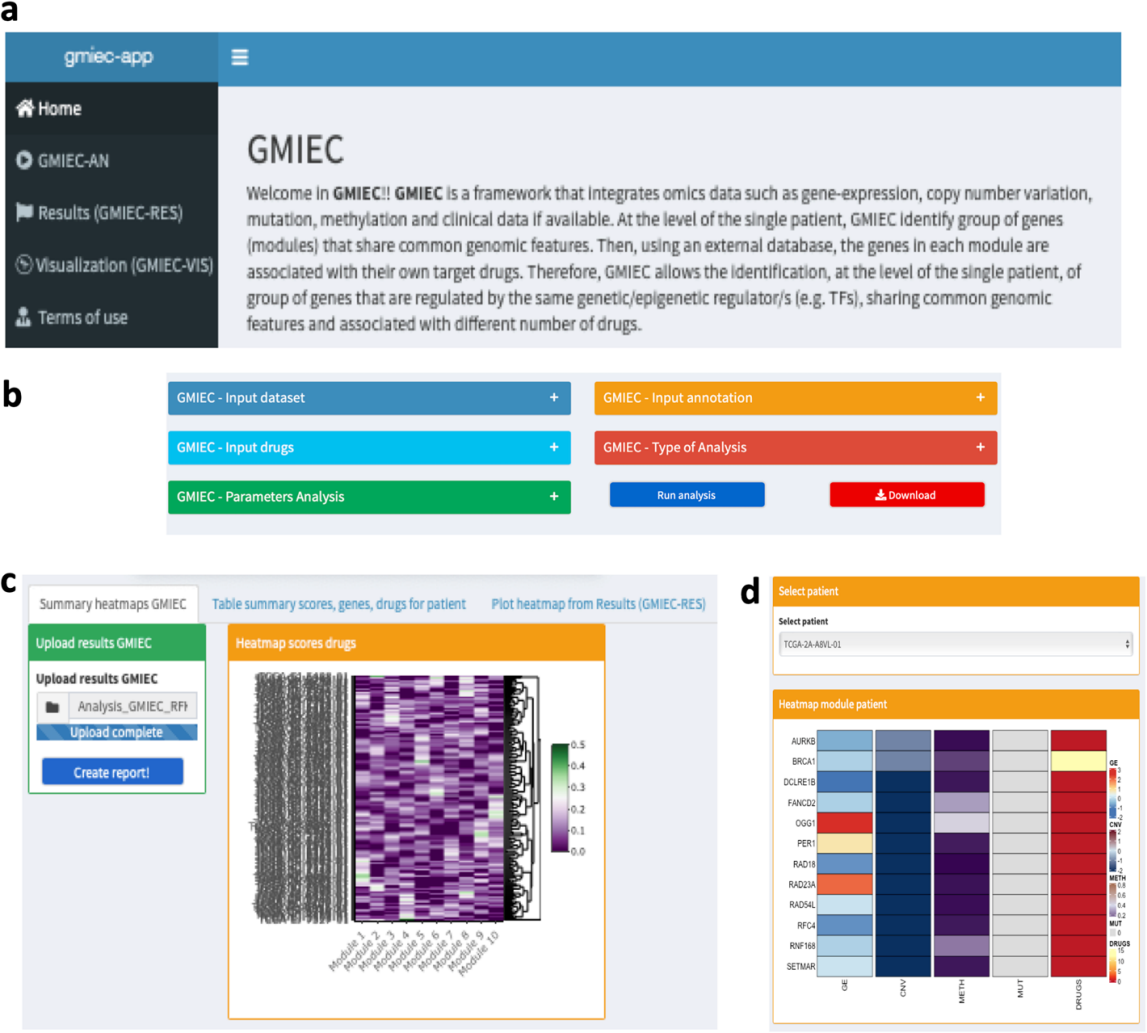
GMIEC interface. **a** The main interface of GMIEC. **b** GMIEC-AN contains fields that support the users to upload the data. **c** Interface of GMIEC-VIS, this application consists of three sub-sections. The first one ‘*Summary Heatmaps GMIEC’* allows the visualization of heatmaps containing the values of drugs, genes, sad or *S-score* ss computed by GMIEC-AN. The second section of GMIEC-VIS allows to inspect the results of GMIEC at the level of single patient with tables. **d** The third section allows to explore the results obtained from GMIEC-RES

GMIEC contains three sub-applications. The user can run the analysis (Fig. 1) using the GUI interface (Fig. 2a) and the “tabs” available:

- **GMIEC-AN:** GMIEC-Analysis (GMIEC-AN) is the principle module of GMIEC and allows to analyze multiple datasets. The user can easily upload the data using dedicated fields and windows (Fig. 2b). These windows allow to select the type of analysis (M1 or M2) and options (see section “Options to run GMIEC-AN”, Additional File 1) to employ.
- **Visualization (GMIEC-VIS)**: is another interface that allows to upload the results of GMIEC-AN and explore the data. This application consists of three sub-sections (Fig. 2b). The first one ‘*Summary Heatmaps GMIEC’* allows the visualization of heatmaps containing the values of drugs, genes, sad or *S-score* values computed by GMIEC-AN. These heatmaps are interactive, the user can zoom in or out to explore the results (Fig. 2b). The second section of GMIEC-VIS allows to inspect the results of GMIEC at the level of single patient. The user can select a given patient to visualize a table that contains several statistics for each module (e.g. number of genes, number of drugs, scores drugs, scores genes, sad or *S-score*). GMIEC-VIS generates also tables with the drugs and genes identified in the module with external links to NCBI and DGIdb. The visualization tab contains also a functionality (Fig. 2c), that facilitates the visualization of the modules, from the simplified version of the GMIEC output table obtained from GMIEC-RES. This sub-application contains an interface similar to those one of GMIEC and the user can upload the result of GMIEC, the omics data and visualize for each patient the gene-expression, copy-number, methylation levels, the mutational status and the number of the drugs for the genes identified in the module (Fig. 2d).
- **Results (GMIEC-RES):** The output obtained from GMIEC-AN can be uploaded into GMIEC-results (see section “GMIEC-results” Additional File 1 for a complete description of GMIEC-RES). This application allows i) the automatic selection of the modules from each patient; ii) the parsing of GMIEC-AN output in a simple form; iii) visualize the results of GMIEC-AN. The concept underlying GMIEC-results is selecting group of the genes in one patient that are the most over-activated (e.g. genes with high copy-number) or over suppressed (e.g. genes hypermethylated) and that are targets of drugs. Although this approach is a simplification, GMIEC-results it is a straightforward approach to support the users with few computational skills to parse the output of GMIEC-AN and easily identify the modules that could be biologically more relevant. More expert users can use the original output file created by GMIEC-AN to select the modules using custom approaches. The output of GMIEC-results is a tab delimited file with the rows corresponding to the patients and four columns with the patient id, the score of the modules, the genes and the drugs inside each module.

### Case study

To illustrate the functionalities of GMIEC, we analyzed omics data of 153 patients with prostate cancer from the TCGA repository and a list of genes involved in DNA damage response (DDR) pathway [22]. This is a pathway that has been identified deregulated in prostate cancer [23]. The main purpose of this analysis (see “Analysis of patients with prostate cancer” Additional File 1, for details of analysis) was the identification of active oncogenic modules (AOMs) associated with drugs (AOMDs) at the level of single patient. This is particularly useful in order to select modules that could be deregulated and associated with drugs on the basis of our scoring system. A number of 123 and 86 individuals were identified respectively with the AOMDs and AOMs with at least one drug. When we explored the results of the patients with AOMDs (see “Analysis of patients with prostate cancer” Additional file 1, Fig. S2), these modules were characterized by several up-regulated genes, gains in their copy number status, low levels of methylation, and mutations. We also selected the inactive oncogenic modules (IOMDs) associated with drugs (see “Analysis of patients with prostate cancer” Additional file 1, Fig. S3). The AOMDs were different between the patients in terms of genes, size (number of genes) and associated drugs (see “Analysis of patients with prostate cancer” Additional file 1, Fig. S2). For example, (see “Analysis of patients with prostate cancer” Additional file 1, Fig. S2B) shows a patient with an up-regulation of the gene ERCC6, which is involved in DNA repair, associated with the molecule cisplatin in a specific patient. Instead, (see “Analysis of patients with prostate cancer” Additional file 1, Fig. S2C) shows another patient who over expresses the gene ATR, which is involved in DNA repair and apoptosis regulation, that is associated with olaparib and temozolomide molecules. These modules might be exploited in future to target the DDR pathway using a personalized approach based on the knowledge of the genetic status of the single patients. The user can also explore the modules containing specifics genes (see section “Analysis of modules containing a specific gene” Additional file 1 and Fig. S4 for details).

## Conclusions

GMIEC allows the identification, for each individual, of group of genes that share common genomic features in order to find putative target drugs. The interface of GMIEC allows the user, with no advanced programming skills, to obtain an output that can be queried in a very easy way. Moreover, GMIEC presents a great flexibility. In particular, it allows the analysis of two or more omics datasets, allowing the users with few data to perform the analysis. GMIEC-AN implements multiple options for the analysis and return as output a simple tab-delimited. Then, this output can be uploaded into two other sub-applications (GMIEC-results and GMIEC-VIS) to facilitate the detection of gene modules and the exploration of the data. The features that, as far as we know, are not present in other software, makes GMIEC a new tool that can be useful in the context of the data integration, data mining and data-exploration at the level of single patient. GMIEC was developed as a tool to use for research purpose only. The authors decline all responsibility on its usage in clinics.

## Availability

**Project name:** GMIEC.

**Project home page:** https://github.com/guidmt/GMIEC-shiny.

**Operating system(s):** Platform Indipendent.

**Programming Language:** R.

**Other requirements:** Dependent on R packages and web browsers (e.g. Chrome, Safari, Firefox).

**License:** MIT.

**Any restrictions to use by non-academics:** None.

## Supplementary information

**Additional file 1.** Supplementary methods and results. This document contains an extended description of the methods implemented in GMIEC, the supplementary results and the supporting Figs. S1-S4.

## Data Availability

The prostate cancer data [24] used to test the functionalities of GMIEC were downloaded from www.cbioportal.org [25, 26]. The datasets generated during and/or analysed during the current study are available in GMIEC repository.

## Abbreviations

GMIEC: Genomic modules identification et characterization for genomics medicine
GMIEC-AN: GMIEC-analysis
GMIEC-VIS: GMIEC-visualization
GMIEC-RES: GMIEC-results
NGS: Next generation sequencing
M1: Method 1
M2: Method 2
vcf: Variant call format
GUI: Graphical user interface
sad: Score alterations and drugs
GMs: Gene modules
DMs: Drug modules
NCBI: National center for biotechnology information
TCGA: The cancer genome atlas
DDR: DNA damage response
AOMs: Active oncogenic modules
AOMDs: Active oncogenic modules associated with drugs
IOMDs: Inactive oncogenic modules
