# Supplementary material for: GMIEC: a shiny application for the identification of gene-targeted drugs for precision medicine
Source: BMC Genomics. 2020 Sep 10;21:619. doi: 10.1186/s12864-020-06996-y (PMC7488130; doi:10.1186/s12864-020-06996-y)
Supplement: Supplementary file 1 — Additional file 1. Supplementary methods and results. This document contains an extended description of the methods implemented in GMIEC, the supplementary results and the supporting Figs. S1-S4. [file 12864_2020_6996_MOESM1_ESM.docx]

GMIEC: a Shiny application for the identification of gene-targeted drugs for precision medicine

Guidantonio Malagoli Tagliazucchi^a,1^, Cristian Taccioli^2*^

^a^ Division of Cardiology, Azienda Ospedaliero-Universitaria di Parma, 43126 Parma, Italy.

^1^ Present address: Department of Genetics, Evolution and Environment, Darwin Building, Gower Street WC1E 6BT London ,

^2^ Department of Animal Medicine, Production and Health, University of Padova, 35020 Legnaro (PD), Italy;.

*To whom correspondence should be addressed.

**Contents**

[**Installation and usage** 2](#_Toc42292673)

[**Prerequisite** 2](#_Toc42292674)

[**GMIEC** 2](#_Toc42292675)

[**I.** **GMIEC framework of analysis** 2](#_Toc42292676)

[**Computational methods** 3](#_Toc42292677)

[**I.** **Method 1 (M1): random forest and k-means** 3](#_Toc42292678)

[**II.** **Method 2 (M2): logic approach and k-modes** 3](#_Toc42292679)

[**Computation of the scores** 4](#_Toc42292680)

[**I.** **S-score** 4](#_Toc42292681)

[**II.** **Scores computed by method M2** 5](#_Toc42292682)

[**III.** **SAD score** 5](#_Toc42292683)

[**Options to run GMIEC-AN:** 5](#_Toc42292684)

[**GMIEC-results** 6](#_Toc42292685)

[**Case study: “Analysis of patients with prostate cancer”** 6](#_Toc42292686)

[**I.** **Analysis of modules containing a specific gene** 9](#_Toc42292687)

[**References** 10](#_Toc42292688)

# **Installation and usage**

GMIEC was developed as a Shiny application. To facilitate the installation of our tool, GMIEC is released with an R script that install all mandatories dependencies. GMIEC was tested on a local laptop with the following characteristics: 12 GB ram, and a processor AMD A10-8700P Radeon R6 Compute Cores 4C+6G on a 64-bit operating system. To analyze the prostate cancer dataset GMIEC-AN module employed about 3 minutes. GMIEC-shiny is available at the following link: <https://github.com/guidmt/GMIEC-shiny>.

# **Prerequisite**

- Install R >= 3.6 .0, available on https://cran.r-project.org/
- for windows user install RStudio (Optional), available on https://www.rstudio.com/products/rstudio/download/
- dependencies R packages: shiny, shinydashboard, klaR, formattable, bcp, plyr, kableExtra, randomForest, heatmaply, ChIPpeakAnno [1], ComplexHeatmap [2], circlize[3]
- To install all these packages, use the installation file provided on <https://github.com/guidmt/GMIEC-shiny>

# **GMIEC**

## **GMIEC framework of analysis**

**Figure S1. Details of GMIEC-AN.** This workflow shows the procedures implemented in GMIE-AN to identify the genes modules. In the first step, the user uploads the genomic data, through a web interface. In this case, the analysis starts with gene expression data and copy number alteration matrices. Moreover, a list of genes it is also provided. In the second step, GMIEC-AN uses the gene-set G to perform a sub-selection of the genomics profiles from the gene-expression (GE) and copy number variation data (CNV). For each subject, GMIEC-AN create a matrix (g x m). The rows of this matrix correspond to the number of genes uploaded. The columns are the omics datasets available, in this case GE and CNV. This matrix is used as input for random forest algorithm (step 4) for the computation of the proximity values. This is the procedure of analysis implemented with M1. Otherwise, a logic rule approach it is used to identify group of genes with similar properties (M2). The output obtained from step 4 it is used as input for step 5. This is the step of the identification of genes modules and it is performed respectively with k-means or k-modes depending on the fact that a user selects the M1 or M2 approach. In step 7 the output of the k-means or k-modes analysis is merged with a file that contains the information of the associations between genes and drugs. The last step of GMIEC-AN it is the computation of the scores for the genes and drugs modules.

# **Computational methods**

GMIEC-AN implements two strategies of analysis. A summary with the differences between these two methods it is reported in Supplementary Table 1.

|  | **Algorithm** | **Two-datasets** | **Multiple-datasets** | **Scoring system** |
| --- | --- | --- | --- | --- |
| M1 | Random forest + k-means | X | X | S-score |
| M2 | Logic rules + k-modes | X | X | LAM, RDG, SAD score |

**Supplementary Table S1**. The main difference between method M1 and M2 is the algorithm used to identify the modules. Both M1 and M2 are able to analyze a minimum number of two omics datasets. Another relevant difference is that M1 compute an S-score, whereas M2 compute a SAD score.

The user can select the methodology and the parameters of analysis (M1 or M2) using a specific “box” (GMIEC-Parameters of analysis) in the GMIEC-AN interface. This window contains a sub-section in which the user can select the parameters to analyze a minimum of two datasets.

## **Method 1 (M1): random forest and k-means**

This method integrates the genomic data and identify the gene modules based on an unsupervised machine learning approach. The algorithm first identifies the subjects in common between all genomic datasets. Then, for each individual, a matrix is generated, in which the rows are the genes considered for the analysis and the four columns contains respectively the gene-expression, methylation, copy-number, and the genetic values. The underlying distributions could different and dependent on the type of data and analytical framework to analyse it. The gene-expression, methylation and copy number data presents different data distributions. The gene-expression data could be log-normal distributed or modelled by Poisson, negative binomial distribution [4]. In the most standard case, the gene-expression data can assume continuous values from 0 to the maximum value of expression. GMIEC-AN codify the presence of a variant of a gene as 1 or 0. The ß-values of the methylation data could be described by bimodal distribution, with ranges between 0 and 1. Finally, copy number data are not normally distributed with ranges that can be between 0 and the maximum value of copy number, or between -∞ and ∞. In dealing this type of data, unsupervised random forest models (URF) is a fast solution , in fact, does not rely on any assumption on the distribution, the scaling, attributes (continuous or categorical) [5], [6]. URF has been applied successfully to handle mixed data type in the context of clinic [7]. URF analysis were successfully applied in the fields of genomic [8],[9], neurology [10]. In M1, when the proximity matrix is computed, the usage of a traditional clustering algorithm such as *k*-means was implemented to identify the modules and perform the partitioning of the data.

## **Method 2 (M2): logic approach and k-modes**

These are the rules applied in all possible settings of GMIEC:

Rule1: if the genes are overexpressed return 1 else 0

Rule2: if the genes are down-expressed 1 else 0

Rule3: if the genes are low-expressed 1 else 0

Rule4: if the genes are expressed 1 else 0

Rule5: if the genes are not expressed 1 else 0

Rule6: I the genes are gain in their copy-number status (*gCNV*):

**if** (*gCNV ≥ 1 & gCNV* < 2*)* return 1 **else** return 0

Rule7: The genes are amplified in their copy-number status (*gCNV*):

**if** (*gCNV* > 2*)* return 1 **else** **if** (gCNV<2) return 0

Rule8: The genes are loss in their copy-number status (*gCNV*):

**if** (*gCNV* > - 2 & *gCNV* ≤ - 1*)* return 1 **else** **if** (gCNV >-1)return 0

Rule9: The genes are depleted in their copy-number status (*gCNV*):

**if** (*gCNV* < - 2*)* return 1 **else** **if** (gCNV >-1) return 0

Rule10: The genes present is hyper-methylated (*gMETH*):

**if** (*gMETH* *≥* 0.5*)* return 1 **else** return 0

Rule11: The genes present is hypo-methylated (*gMETH*):

**if** (*gMETH* ≤ 0.5*)* return 1 **else** return 0

Rule12: If a gene is mutated (gMUT):

**if** (gMUT is mutated*)* return 1 **else** return 0

# **Computation of the scores**

## **S-score**

The $S-score$ [11], quantifies if a group of genes are potentially over activated (oncogenic) or suppressed. The genes are defined as oncogenic if presents amplifications (*Namp*) in their copy number status or are over-expressed (*Noe)*. Genes with high level of methylation (*Nmet*), depletion (*Ndel*) in their copy number status, presence of mutations (*Nmut*), or under-expressed (*Nue)* are defined as suppressed. This score is particularly suit in the case of the analysis of cancer data. For other types of diseases, the user can consider as over-activated the genes that are considered ‘oncogenic’ according to the *S-score*. For the genes that are repressed the interpretation does not change. The computation of this score is available only for GMIEC-AN. A similar scoring system was described in. The only differences between the original formulation of the $S-score$ and those one implemented in our tool it is that GMIEC-AN compute also the number of genes that are over and under expressed in a gene module. Here the definition of the components to compute the $S-score$.

1. *Namp:* is the number of genes with amplification in their copy number status. All genes with a copy number ≥ of 1 are considered amplified.
2. *Noe:* is the number of genes that are over-expressed.
3. *Nmet*: number of genes in which their respective genes are methylated. The threshold implemented to detect methylated genes is 0.7.
4. *Ndel:* is the number of genes with deletions in their copy number status. All genes with a copy number ≤ of -1 are considered depleted.
5. *Nmut*: it is the fraction of genes with mutations.
6. *Nue:* is the number of genes that are under-expressed.

To compute the *Noe, Nue* values GMIEC-AN transform the gene-expression values in z-scores. All genes with an expression ≥ 1.5 are considered over-expressed. Genes with an expression < 0 are considered under-expressed.

After this step, GMIEC-AN compute a SAD score, this score takes in account the number of drugs that target a group of genes and the number of alterations in these genes (see section SAD score). In the case that no drugs target a group of genes this score becomes 0. In this case the user can take only in consideration the S-score.

## **Scores computed by method M2**

When GMIEC-AN is run with method ***M2*** the computation of the SAD score is performed differently (see section *Computation of the scores* for details). With this analysis the user can identify group of genes that show genomics alterations. Because ***M1*** suites well with data from cancer, ***M2*** can be applied to analyze dataset from other diseases. However, similarly for ***M1***, also in this case will be possible capture genomic differences between patients and therefore identify targets of pharmacological treatments. The modules defined by GMIEC-AN with **M2** consist of **n** rows, corresponding to the number of genes and **r** columns (as the number of rules defined). We define ***c*** as the cell of n_i_ row and r_i_ column. For one gene (**g**), ***c*** can assume binary value (0,1) indicating the presence or not of a genomic property (*See section Method 2: logic approach + k-modes*). In this context, we will use the term “alterated module” to indicate a group of genes with some genomic alteration. In parallel to this, we identified also if a module is druggable or not. A module highly druggable is a module in which many genes are targeted by drugs. Finally, a unique score indicates the levels of alterations and the “druggability” of a module.

The total size of a module (tsm) is given by:

$$Total size module \left( tsm \right)=n \times r$$

To estimate the levels of alteration we computed ratio between the number of ***c*** with or without the alteration and the *tsm.* In detail:

For each **m** estimate the number of ***c*** with alteration (***c***_a_) to get a “level alteration module” (*LAM*).

$$LAM= \frac{\#\boldsymbol{c}a}{tsm}$$

Then, considering a module, the calculation of the number of genes targeted by drugs or not is estimated. Then, for each **m,** the ratio between the number of ***g*** targeted by drugs (***g***_tg_) and the total number of genes in module (***t***_gm_) is performed. This leds to obtain the ratio of druggable genes for module (RDG).

$$RDG= \frac{\boldsymbol{g}\mathrm{tg}}{\boldsymbol{t}\mathrm{gm}}$$

## **SAD score**

In the case that one analysis is performed with M1, the SAD score is:

$$SAD= S-score \times RDG$$

When a GMIEC analysis is run with M2, the SAD score is:

$$SAD= LAM \times RDG$$

# **Options to run GMIEC-AN:**

GMIEC-AN can be run with different options:

1. “***Use only the genes annotated***”: with this option the user can upload the omics data, the annotation file, and a file with the genomic coordinates (e.g. bed file). Only the genes that are annotated in the bed file are used for the analysis.
2. “***Use all genes***”: with this option the user must upload the omics data. GMIEC-AN will consider all common genes between the different datasets.
3. “***Use a list of genes***”: with this option can provide a custom gene list for the analysis.

# **GMIEC-results**

GMIEC-results implements four strategy that prioritizes the modules of each patient based on the maximum or the minimum values of *S-score* and SAD score:

- Module active: in this case GMIEC-results selects, from each patient, only the module with the highest score *S-score.* This method can be useful to detect the most active groups of genes in one patient.
- Module inactive: GMIEC-results in this modality selects, from each patient, only the module with the lowest score *S-score*. This approach can be useful to detect the most inactive groups of genes in one patient.
- Module active with drugs: GMIEC-results selects, from each patient, only the module with the maximum SAD score. This method can be useful to detect the most inactive groups of genes in one patient.
- Module inactive with drugs: GMIEC-results selects, from each patient, only the module with the lowest SAD score. This method can be useful to detect the groups of inactive genes associated with drugs.

In all cases the resulting modules will be associated with the annotated drugs. The differences between *S-score* and SAD score is that in the first case the user can find the most deleterious modules independently by the number of drugs associated. Instead, when GMIEC-results selects the modules considering the SAD scores, only the modules with the highest number of associated drugs will be searched.

# **Case study: “Analysis of patients with prostate cancer”**

1. **Materials:**

GMIC-AN was tested on a real dataset of 153 patients with prostate cancer with a diagnosis of prostate cancer (1-ERG subtype) downloaded from TCGA (Cancer Genome Atlas Research Network, 2015). This dataset consists of gene-expression, copy-number variations, methylation and mutation data. Because GMIEC-AN requires as additional input, a file that reports the associations between the genes and drugs, we created a custom file using DGIdb [12] and selecting only the drugs known to be used in clinic to treat cancer. A list of 193 genes important in DNA repair were downloaded from [13]. Prostate cancer data were download from TCGA [14]. The analysis was performed using GMIEC-AN using M1 (random forest + k-means). A number of 8 clusters were used to group the genes.

1. **Analysis details:**

The analysis was performed using GMIEC-AN using M1 (random forest + k-means). A number of 8 clusters were used to group the genes. The main purpose of this analysis was the identification of active oncogenic modules (AOMs) associated with drugs (AOMDs) at the level of single patient. A GM consist of a group of genes with similar genomic characteristics (e.g. gene-expression and copy number variation levels).

1. **Supplementary results:**

**Figure S2.** **GMIEC-results output, heatmaps of AOMDs modules.** This figure contains the results of GMIEC-AN and GMIEC-results for 9 patients. For some patients we found that the modules contain up-regulated genes (e.g. a-d). We also identified also patients with up-regulated genes and gain in their copy number status (e.g. Fig e-g). Few levels of methylations and mutations were observed in the considered modules.

**Figure S3.** **GMIEC-results output, heatmaps of IOMDs modules.** This figure contains the results of GMIEC-AN and GMIEC-results for 8 patients. For some patients we found that the modules contain down-regulated genes (e.g. c, e, g). We also identified also patients with down-regulated genes and depletion in their copy number status (e.g. e). In one patient we found a module with mutated genes (d). High levels of methylations were observed (e.g. a, b, c, f, h).

## **Analysis of modules containing a specific gene**

The user can also identify, the modules containing a given gene. For example, we explored all the AOMDs modules containing BRCA1 which is an important gene of DDR pathway. In this way, we identified 12 subjects showing this gene deregulated (Fig. S10l-S10M). Finally, our analysis allowed to detect modules at level of single patient, the drugs that are validated molecules often used in clinics, and the patients harboring the mutated BRCA1 gene.

**Figure S4.** **GMIEC-results output, example of patients with different expression of BRCA1.** For each patient carrying an AOMD with BRCA1, the figure describes the gene-expression, copy-number, methylation, mutation data. The drugs associated with each gene are depicted in the other column.

# **References**

1. Zhu LJ, Gazin C, Lawson ND, Pagès H, Lin SM, Lapointe DS, et al. ChIPpeakAnno: a Bioconductor package to annotate ChIP-seq and ChIP-chip data. BMC Bioinformatics. 2010;11:237.

2. Gu Z, Eils R, Schlesner M. Complex heatmaps reveal patterns and correlations in multidimensional genomic data. Bioinformatics. 2016;32:2847–9.

3. Gu Z, Gu L, Eils R, Schlesner M, Brors B. circlize implements and enhances circular visualization in R. Bioinformatics. 2014;30:2811–2.

4. Pachter L. Models for transcript quantification from RNA-Seq. arXiv:11043889 [q-bio, stat]. 2011. http://arxiv.org/abs/1104.3889. Accessed 3 Jun 2020.

5. Shi T, Horvath S. Unsupervised Learning With Random Forest Predictors. Journal of Computational and Graphical Statistics. 2006;15:118–38.

6. Unsupervised random forest: a tutorial with case studies - Afanador - 2016 - Journal of Chemometrics - Wiley Online Library. https://onlinelibrary.wiley.com/doi/10.1002/cem.2790. Accessed 29 May 2020.

7. Ngufor C, Warner MA, Murphree DH, Liu H, Carter R, Storlie CB, et al. Identification of Clinically Meaningful Plasma Transfusion Subgroups Using Unsupervised Random Forest Clustering. AMIA Annu Symp Proc. 2018;2017:1332–41.

8. Alhusain L, Hafez AM. Cluster ensemble based on Random Forests for genetic data. BioData Mining. 2017;10:37.

9. Derkarabetian S, Castillo S, Koo PK, Ovchinnikov S, Hedin M. A demonstration of unsupervised machine learning in species delimitation. Molecular Phylogenetics and Evolution. 2019;139:106562.

10. Cao L, Li J, Zhou Y, Liu Y, Zhao Y, Liu H. Online identification of functional regions in deep brain stimulation based on an unsupervised random forest with feature selection. J Neural Eng. 2019;16:066015.

11. de Souza JES, Fonseca AF, Valieris R, Carraro DM, Wang JYJ, Kolodner RD, et al. S-score: a scoring system for the identification and prioritization of predicted cancer genes. PLoS ONE. 2014;9:e94147.

12. Cotto KC, Wagner AH, Feng Y-Y, Kiwala S, Coffman AC, Spies G, et al. DGIdb 3.0: a redesign and expansion of the drug-gene interaction database. Nucleic Acids Res. 2018;46:D1068–73.

13. Chae YK, Anker JF, Carneiro BA, Chandra S, Kaplan J, Kalyan A, et al. Genomic landscape of DNA repair genes in cancer. Oncotarget. 2016;7:23312–21.

14. Cancer Genome Atlas Research Network. The Molecular Taxonomy of Primary Prostate Cancer. Cell. 2015;163:1011–25.
